# Hearing Aid Fitting in Tinnitus: A Scoping Review of Methodological Aspects and Effect on Tinnitus Distress and Perception

**DOI:** 10.3390/jcm10132896

**Published:** 2021-06-29

**Authors:** Dimitrios Kikidis, Evgenia Vassou, Nikolaos Markatos, Winfried Schlee, Eleftheria Iliadou

**Affiliations:** 1 First Department of Otorhinolaryngology, Head and Neck Surgery, National and Kapodistrian University of Athens, Hippokration General Hospital, 11527 Athens, Greece; evassou@med.uoa.gr (E.V.); markatosn@med.uoa.gr (N.M.); iliadoue@med.uoa.gr (E.I.); 2 Department of Psychiatry and Psychotherapy, Universität Regensburg, 93053 Regensburg, Germany; winfried.schlee@gmail.com

**Keywords:** tinnitus, hearing aid fitting, tinnitus perception

## Abstract

Current evidence on efficacy of hearing aids (HAs) on tinnitus perception and annoyance is considered insufficient due to the heterogeneity of tinnitus characteristics and of methods used in the relevant clinical studies. This is a scoping review focused on the methodological aspects of clinical studies evaluating the value of HA fitting as part of tinnitus management over the past 10 years. Thirty-four studies were included in the review, showing important heterogeneity in almost all aspects of inclusion criteria, comparators, outcome measures, follow-up time and HA fitting procedures. Although all studies show that HA fitting has a positive impact on tinnitus perception in patients with hearing loss, the methodological heterogeneity does not allow robust conclusions. Future studies taking into account the different nature and goals of each tinnitus therapeutic modality and adapting their methods, endpoints and timelines according to them could lay the groundwork for obtaining high-quality evidence on whether and how HA fitting shall be implemented in tinnitus management strategies.

## 1. Introduction

Tinnitus is traditionally defined as the perception of a sound in the absence of external stimuli; however, this definition has recently been updated in order to include patient’s reaction and related annoyance as a determining factor [1]. Universally effective and accepted tinnitus treatment is currently pending, although a long list of substances and interventions, including but not limited to medicinal agents, sound treatment (ST), Transcranial Magnetic Stimulation (TMS), acupuncture and hearing amplification, has been tested for their efficacy in multiple studies [2].

Hearing loss and tinnitus are highly correlated, since it is estimated that up to 90% of patients experiencing tinnitus suffer from various degrees of hearing loss as well [3]. However, degree of hearing loss is not established as a prognostic factor for tinnitus existence and annoyance [4,5]. On top of this, approximately 10% of individuals with tinnitus have normal thresholds in Pure Tone Audiometry (PTA) [6]. This fact has triggered a wide interest in the literature with regards to cochlear synaptopathy which corresponds to a possible pathophysiological feature causing loss of the low spontaneous rate (low-SR) synapses without elevation of the PTA thresholds, as initially proposed by Schaette et al. (2011). Nevertheless, this concept has been recently questioned [7,8,9].

According to recent European guidelines, there is a weak recommendation for the hearing aids (HAs) in tinnitus treatment [1]. It is also stated that tinnitus presence should be taken into account during the hearing aid fitting procedure. However, supporting literature has been characterized as inadequate to draw certain conclusions, due to lack of relevant high-quality studies [1]. Hence, it should be highlighted that, although Randomized Clinical Trials (RCTs) are being considered as the best source of high-quality data, this study design is probably not applicable in the context of hearing aid fitting, since participants of control groups would immediately understand that they are provided with some kind of sham device. HAs mainly target hearing loss, their main effect cannot thus remain unnoticed. This is an intrinsic drawback that is very difficult to overcome [10]. Apart from these study design aspects, the primary and secondary endpoints that are chosen to assess the success or not of the intervention (hearing aid fitting) should be evaluated, since they may interfere with the quality of the results [11]. In the case of hearing aid fitting, commonly used outcome measures cannot reliably reflect the elimination of tinnitus that happens in a robust subgroup of patients.

A review and critical appraisal of published evidence on methodological aspects and results of clinical studies focusing on the effect of HA fitting on tinnitus perception could provide valuable insight for future studies and clinical practice.

## 2. Materials and Methods

This paper is a scoping review of the literature, aiming at pointing out the effect of hearing aid fitting on tinnitus perception and the methodological aspects of the relevant clinical studies. It is following the PRISMA Extension for Scoping Reviews (PRISMA-ScR) guidelines [12].

The main goals of this paper were to identify studies that describe hearing aid fitting in the case of people with tinnitus and evaluate their methodology as well as the effect of hearing aid fitting in tinnitus perception and related handicap, distress, annoyance and loudness. 

Review questions were set as following: What are the methodological aspects of stu-dies evaluating the effect of HAs fitting on tinnitus perception? Is there an effect of the various HA fitting devices and methods on the perception of tinnitus characteristics in adults with hearing loss? More specifically, the question was formulated according to the PICO template as following:

People: adults with tinnitus (bothersome or not) and hearing thresholds requiring amplification or not.

Intervention: hearing aid fitting with or without use of maskers and specific fitting techniques. 

Comparator: not applicable.

Outcomes: methodological aspects such as range of included hearing loss or outcome measures used and effect of HA fitting on tinnitus handicap, distress, annoyance and loudness as reported in questionnaires and scales used as outcome measures before and after hearing aid fitting.

### 2.1. Eligibility Criteria

Studies were selected according to the following criteria:

Study Samples: Target population consists of adults with tinnitus, with hearing thresholds requiring amplification or not. Particulars: There was no restriction in tinnitus type. Studies that had a primary goal other than evaluating tinnitus were not excluded, as long as the results of hearing aid fitting on tinnitus perception were reported. There was no restriction in types of hearing loss. Sudden hearing loss, age-related hearing loss, noise trauma, hereditary hearing loss, otosclerosis etc, were all included to the review.

Intervention: hearing aid fitting, (no limitation on particular methodology, fitting technique, laterality, manufacturer or equipment).

Clinical experimental studies, case reports, case series, observational studies (longitudinal and cross-sectional), methodological papers, randomized clinical trials were included. Studies conducted during the past 10 years have been chosen for inclusion; stu-dies conducted with focus on hearing aid technology older than that has been considered as out of the scope of this review. On-going studies, pediatric population studies, cochlear implantation and Tinnitus Retraining Therapy (TRT)-related studies, reviews and meta-analyses, experts’ opinions and letters to the editor were all excluded. Articles in a language other than English were also considered non-eligible for this review.

### 2.2. Information Sources

Four major databases (Medline, Central, Web of Science, ClinicalTrials.gov (accessed on 19 May 2021) and Scopus) have been searched for eligible studies by two reviewers independently. The results were then hand-searched [13].

### 2.3. Search

Typically, literature search includes three sets of terms: terms concerning the health condition of interest (in our case, tinnitus), terms describing the intervention/exposure (Hearing aid fitting) and terms for the type of eligible studies (not applicable in our case since we have no particular limitation in study type) [14]. In this context, the search syntax for this scoping review for Medline was:

(amplification OR “hearing aid” OR (“hearing aids”[MeSH]) OR “hearing aid fitting”) AND (tinnitus OR tinnitus[MeSH])

The rest of the databases have been searched in a similar manner, using the same keywords. Filters of “10 years” and “English” language have been applied in all databases. Filters excluding non-clinical studies and pediatric studies have been applied whenever available. The whole search procedure and results have been evaluated by means of PRESS Evidence-Based Checklist [15].

### 2.4. Selection of Sources of Evidence

Studies obtained from the aforementioned search were reviewed independently by two authors. In that stage of analysis, the authors identified duplicates or multiple reports of the same study. Then, they screened the relevance of yielded studies to the set research questions by first examining the titles and abstracts of the yielded studies and then their full text. No disagreements between the two authors occurred at this stage.

### 2.5. Data Charting Process

Two reviewers screened full-text articles and produced a matrix of relevant data independently [16]. Ambiguities on data charting have been discussed and resolved by the senior authors.

### 2.6. Data Items

Extracted data items concerning methodological aspects and results of the included tinnitus studies:

- Main author, year of publication
- Sample size
- Whether specific age range was stated as inclusion criterion (Yes/No) and if yes, the actual range
- Whether tinnitus was identified as primary complaint of the participants in the inclusion criteria (Yes/No)
- Range of hearing loss as inclusion criterion (Yes/No)
- Research hypothesis
- Software used for HA fitting
- Whether it was stated that counseling on hearing aid and specific counseling on tinnitus was provided
- Fitting procedure on hearing aid fitting
- Fitting formula
- Number of visits needed for the HA fitting
- Use of masking sound or not
- Treatment of the control arm if existent
- Number of follow-up visits targeting evaluation of the intervention and their time course
- Outcome measures used and whether there was a defined primary outcome measure
- Evidence of improvement (according to corresponding outcome measure)

### 2.7. Synthesis of Results and Critical Appraisal of Individual Resources of Evidence

Results of this scoping review are presented in the form of comprehensive tables. Detailed qualitative analysis and critical appraisal of included studies can be found in the Discussion section. 

## 3. Results

### 3.1. Selection of Sources of Evidence

**Figure 1:**
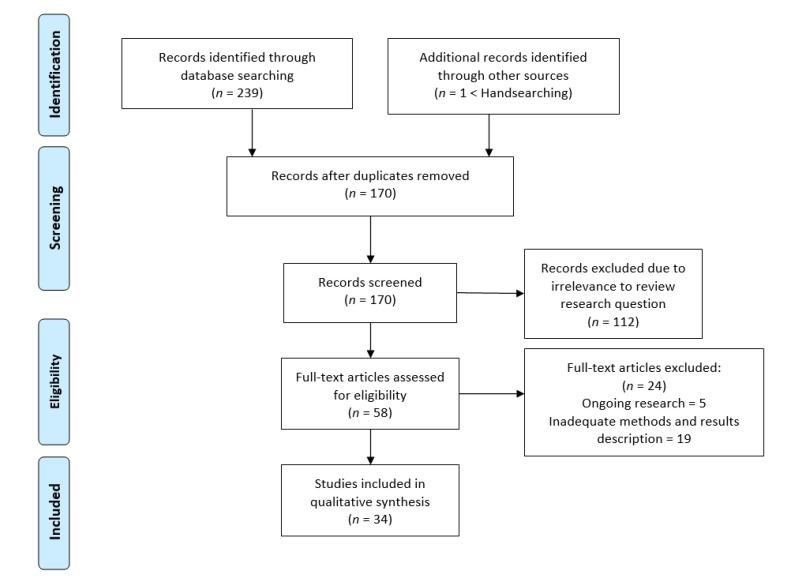
Study selection PRISMA flow diagram.

Thirty-four studies were included in this scoping review. The process of their selection is provided in detail in Figure 1. 

### 3.2. Characteristics of Sources of Evidence and Synthesis of Results

Characteristics of each study, such as authors’ names or year of publication, along with extracted data with regards to the methods used, are presented in Table 1, Table 2 and Table 3. Inclusion criteria (hearing loss range, tinnitus duration, tinnitus as primary complaint) and types of participants’ groups are presented in Table 1. Fitting methods and whether tinnitus-specific counseling has been provided can be found in Table 2, while results of each study with regards to their effect on tinnitus perception in Table 3. A comprehensive list of the methodological limitations of the clinical studies included in this scoping review may be found in Table 4.

## 4. Discussion

This scoping review aimed to summarize current evidence on efficacy of HA fitting on tinnitus characteristics and patients’ annoyance, along with the methodologies the relevant studies have used.

### 4.1. Primary Methodological Aspects of Included Studies

Half of the studies included in this review had less than 40 participants, only three had over 100, whereas there was only one large scale retrospective audit with 974 participants, comparing HAs and sound generators (Table 1). No sample size calculation is described in any of the studies. In addition, no power calculation was provided, neither ad hoc or post hoc. 

A vast majority of studies (25 out of 34) did not set strict age criteria (Table 1). This fact might have an effect on the results since groups might not be adequately heterogenous. Although HL is far more common in older adults, three of the studies with certain age range as inclusion criterion set an upper limit (two of them 70 and one 80 years). Acceptance rates of HA are lower in younger adults and this could lead to lower representation of younger individuals compared to their actual proportion among tinnitus patients. On top of this, older adults might not be familiar with modern technologies recently implemented in HAs, like mobile applications and this could lead to selection bias and higher rates of drop outs, or sub-optimal use in terms of duration. None of the studies, even those who set a certain age range, took these potentially determining factors into consideration. 

Only seven out of 35 studies (20.5%) were RCTs, a fact which highlights the relatively low level of evidence in the field. Eleven were case series with comparisons before and after treatment, whereas 13 were case control studies. Out of these, 5 compared different types of HA fitting, investigating the effect of additional or different features to tinnitus (open vs. classical fit, spectrally notched vs. unmodified, frequency transposition, addition of a sound generator or targeted counseling related to HA fitting), whereas 7 compared effect of HAs with various types of interventions (noise generators, maskers, TRT, notched environmental sound) (Table 1). One of the studies examined the effect of HA aids in two different patient groups, with high and low tinnitus pitch [43]. Another study examined a “prototype” of 3-D masking [39].

Only eight out of 34 studies have clearly set tinnitus as a primary complaint as a certain inclusion criterion (Table 1). Hence identified as a drawback in tinnitus studies, a strict prerequisite of tinnitus as a primary complaint might not be absolutely relevant in studies targeting HA effectiveness in tinnitus. In patients with HL as a primary complaint, hearing aid fitting is indicated anyway. At the same time, a subgroup of patients with tinnitus as a primary complaint, also suffers from HL adequate to set a HA. In real conditions, there is a wide range of importance perception and level of annoyance correlated, between the two poles of HL and tinnitus. This means that patients belong to a wide spectrum between hearing loss and tinnitus as primary complaints—and all the shades in between. This of course cannot eliminate the possibility of patients with HL as a primary complaint hence with a considerably bothersome tinnitus, even catastrophic, or vice versa: patients could mention tinnitus as a primary complaint and at the same time have important communication barriers due to severe hearing loss [51,52]. In conclusion, tinnitus as a primary complaint is of limited value in studies evaluating HA effectiveness in tinnitus, compared to studies targeting other interventions.

Related with this issue is the range of HL suitable for study inclusion. Although all but one studies reported that they targeted patients with HL requiring amplification, only six clearly determined specific thresholds per frequency as inclusion criteria (Table 1). Eighteen of the studies provided some generic HL degrees (mild, moderate, severe), with a large heterogeneity, especially in regards to sever HL. Eleven studies did not have neither a broad determination of HL range, either with absence of any relevant information or with statements like “any type of HL” or “significant loss to warrant HA fitting”. One study included only adults with hearing thresholds not requiring amplification [30]. Potential issues of this broad definitions are twofold. Different types of HAs are optimal for different types of HL. In most of the studies without specific HL inclusion criteria it can only be assumed that the relevant rules are applied, since a relevant statement is not made. Moreover, even if this was valid, it could lead to methodological discrepancies.

Less than half of the studies (14 out of 34) set clear inclusion criteria for tinnitus duration at the time of fitting (Table 1). This could potentially cause a difficulty to estimate effectiveness and clearly sets a potential selection bias. Absence of strict range of tinnitus onset is a draw of tinnitus literature in general, however in the evaluation of the HA effect, this might be even more influential, since there is a considerable proportion of patients who present total or intermittent elimination of tinnitus, which is not usually the case in other types of interventions. It is unknown though, whether a longer tinnitus duration might make more difficult tinnitus elimination or vice versa. In addition, none of the studies used tinnitus onset as a prognostic factor.

### 4.2. Hearing Aid Fitting Procedure

Only 12 out 34 studies gave a clear reference of the fitting formula used (Table 2). The vast majority used NAL (5 used NAL-NL1 and 3 used NAL-NL2), whereas 3 used DSL. This parameter could be potentially important in regards to tinnitus suppression, on top hearing loss amplification, given that different formulas provide different gain patterns. Hence, it is interesting that no study presents a justification about the selected formula nor a predefined hypothesis that one might be more effective on tinnitus compared to another.

With regards to counseling, it is considered the cornerstone of most tinnitus treatments [53]. Majority of the studies included in this review do not describe what type of counseling was included in participants’ workflow or whether they provided any counseling at all. Taking into account the confusion existing among counseling solely for HA fitting, counseling as part of TRT, long-term counseling through Cognitive Behavioral Therapy approaches and actual structured counseling targeting tinnitus, it is evident that the absence of this particular information in the included studies create a significant methodological limitation. Indeed, this is reflected in our results, as well. Only two studies present a clear description of the structured counseling they have conducted; Rocha et al. (2018) reported structured counseling using materials with videos and illustrations proposed by Siemens Audiology Solutions through counseling ‘‘Counseling Suite3.3”. Newman and Sandridge (2012) also presented a detailed list of topics addressed during participants’ education sessions. The rest of the studies reported some kind of counseling, mostly use components of TRT [23,26,33], while three of them do not provide any information [31,32,35]. Commercially available material which is integrated to specific hearing assistive devices (such as Widex Zen therapy) was also used in two studies, however it should be taken into account that this type of counseling deviates from the standard tinnitus counseling conducted by clinicians and its reproducibility is by default limited [40,46]. 

### 4.3. Tinnitus Assessment Methods

According to recent recommendations, outcome measures should be carefully chosen in tinnitus-related clinical studies, depending on the type of intervention. As for HAs, intrusiveness, sense of control, concentration and quality of sleep were among the dimensions that should be targeted by the outcome measures [52].

A sole outcome measure was used in 16 of the studies, whereas the rest used from two up to four outcome measures, with a moderate variance, since nine evaluation tools including validated questionnaires and scales were used in total. Most of the studies (19/34 or 55.9%) used Tinnitus Handicap Index (THI) as a primary outcome measure (Table 3), five Tinnitus Functional Index (TFI) [39,40,41,42,46], one Tinnitus Reaction Questionnaire (TRQ) [29] and two Tinnitus Handicap Questionnaire (THQ) [31,38]. Four studies used more than 2 scales [42,45,46,47]. Scales were used as secondary outcome measures in nine studies and THQ, TQ and THI in two studies each (Table 3).

Although the selected outcome measures could be evaluated as satisfactory, given that they are both in line with the rest of the literature and with the recent recommendations, the main point in regards to evaluation is that these well-established tools are not designed for an intervention that has a far more binary nature compared to the rest. There is a considerable proportion of patients that experience total or close to total tinnitus elimination, at least during HA usage during the day. Questionnaires might globally reflect the change in quality of life, daily function or emotion due to these changes. The opposite could be valid for non-responders.

An additional limitation of currently used methods is that metrics targeting correlation between HA usage and tinnitus suppression in the time domain, usage duration and effect on tinnitus, comparison of HA usage individuals with and without tinnitus as well as some more trivial aspects like tinnitus relapse after HA removal and its effect are still missing. Ecological momentary assessment with use of mobile devices could be a very interesting research field towards this direction. Finally, the effect of total elimination is not well weighted though it might be the case that some patients respond very well and some not at all (binary response).

### 4.4. Follow-Up Period

Table 3 clearly shows that participants’ follow up timeline ranged a lot across included studies. The majority of studies had a follow up time commonly used in the literature (13 of them had 3 months and 5 had 6 months). Only 1 study had a follow up period more than 12 months [28], whereas 3 had a follow up time less than 3 months (ranging from 3 weeks to 2 months) [39,43,44]. These latter are not considered adequate to draw conclusions in the context of tinnitus studies in general, however the effect of hearing aids is not as latent as in other interventions like sound therapy and CBT. On top of this, HA effect on tinnitus has two contradictory characteristics in the time domain: it is intermittent during the day, depending on whether the hearing aid is used or not and on the other hand, it is continuous in the largest time scale, since typically the effect, if present is not expected to substantially change during the period of HA usage. However, it is impressive that none of the studies report any evaluation of these parameters (effect during the day and stability of long-term effect). This latter could be an interesting research question for future studies. 

### 4.5. Results

Before being able to interpret the results of clinical studies comparing two different tinnitus therapeutic modalities, one should take into account that different outcome measures may be more suitable for specific types of tinnitus treatment than others. In the context of COMiT’ID study, Hall et al. (2018) identified and reported the widest approved outcome measures for clinical trials of Sound-, Psychology-, and Pharmacology Based interventions for chronic subjective tinnitus. In the case of HA related clinical studies, COMiT’ID suggests that the minimum set of outcome measures should contain the following: ‘‘ability to ignore’’, ‘‘concentration’’, ‘‘quality of sleep’’ and ‘‘sense of control’’, while psychology-based ones should include endpoints such as “mood”. Although, “what” each clinical study should include as endpoint is clearly stated, to date, no consensus on “how” this endpoint should be obtained and compared between treatment groups exists [11]. In clinical studies comparing a HA fitting with CBT-based therapy, a valid approach would be the inclusion of endpoints relevant to both treatments and the estimation of the effect of each treatment separately. This approach may enable comparisons between treatments in a more binary way, where each treatment has failed or succeeded at creating a significant tinnitus benefit with regards to its corresponding outcomes.

Studies included in this review seem to have adequately covered all the aforementioned core outcome domains through validated tools such as THI or through VAS. However, the same tools are used for all different types of treatment under evaluation, so the interpretation and generalization of their results should be done with caution.

The first important question that has direct research and clinical implications is whether there is evidence that HA fitting may have positive impact in tinnitus perception, distress or annoyance and if yes, what the possibility of improvement that should be expected after HA fitting is. Previous systematic review on HA fitting effect in terms of tinnitus benefit in adults with hearing loss and tinnitus concluded that only one clinical study was of adequate quality and thus no safe conclusion could be reached [54]. 

According to the outcome measures used, HA fitting-related improvement was up to 50%, while the proportion of participants showing at least some improvement ranged from 40% to 85% (Table 3).

As for the effectiveness, a clear trend can be identified among the case series included. All studies claimed a persistent significant improvement, regardless of the type of HA provided and the outcome measures used. Consistency of this finding among eleven studies is important, however it should be evaluated taking into account, on top of low methodological level and heterogeneity, that two of them did not conduct statistical analysis [21,31]. Moreover, one study shows that this improvement may elapse if patients abandon their HA. 

In regards to superiority, only two case control studies compared hearing aid fitting with other types of interventions., namely TRT [33] and counseling [38]. In the latter study, HA fitting was claimed to be superior, however this obviously cannot be considered adequate evidence to draw conclusions. The rest of the case control studies compared either different types of devices and fitting techniques or HA fitting alone versus some kind of maskers or sound generators. 

Whether HA fitting alone is inferior to HA fitting and sound therapy combined still remains unclear. Some studies have shown no statistically or clinically significant difference between HA and combined therapy [22,26], whereas others concluded that the overall benefit was significantly higher in those patients having undergone HA fitting and sound masking [24,25,40,44]. Finally, Jalilvand et al. (2015) showed that amplification alone was superior to sound therapy by means of a noise generator, while Newman and Sandridge (2012) compared sound therapy with broad band noise (BBN) and with Neuromonics Tinnitus Treatment (NTT) observing no statistically significant difference. 

This review has identified seven RCTs, out of which three evaluated the additional effect of sound generator, maskers and fitting techniques (frequency transposition) [22,26]. In none of these trials an additional effect of these interventions was concluded. Other RCTs compared HA fitting against gingko biloba and motivational interviewing [35,49], hence their findings were contradictory and the superiority could not be established.

To conclude, hearing aid fitting, itself, should be considered a valid tinnitus management approach for patients with HL. Current evidence implies that the size of its effect is clinically non-negligible. However, whether it should be combined with sound therapy or not needs further investigation through large scale longitudinal controlled studies. Future studies should overcome the heterogeneity of tinnitus assessment tools and outcome measures used, the different follow-up timelines and the conflicts of interest in those studies using commercially available tools so that they can lead safely to robust conclusions. Future studies with adequate study design and sample sizes, clearly set demographic inclusion criteria, clear ranges of hearing loss and tinnitus characteristics of the included subjects are warranted.

## Figures and Tables

**Table 1:** Primary methodological aspects of included studies.

ID	Study Design	Participants No	Age	Hearing Loss (HL) Range	Tinnitus Duration	Tinnitus Being Primary Complaint	Groups
Acar, 2014 [17]	Case Series	24	>65 years	Sufficient HL to warrant the use of Hearing Aids (HAs)	Not determined	Yes	HA fitting
Araujo, 2016 [18]	Case Series	24	60–70 years	41–60 dB HL at 500, 1000, 2000, and 4000 Hz	4–30 years	No	HA fitting Tinnitus Group vs. HA fitting without Tinnitus Group
Berberian, 2016 [19]	Case Series	25	No	Mild to moderately severe HL	Not determined	No	HL and bilateral tinnitus
Cabral, 2016 [20]	Case Series	17	No	Mild to severe sensorineural HL (SNHL) or mixed HL	Not determined	Yes	HA fitting
Cribari, 2016 [21]	Descriptive cross-sectional study	53	>60 years	SNHL or mixed HL, moderate, moderately severe, severe	Not determined	No	HA fitting
dos Santos, 2014 [22]	Randomized Control Trial (RCT)	49	No	Mild to moderate bilateral symmetrical SNHL	>6 months	No	Combined fitting group vs. amplification alone group
Forti, 2010 [23]	Case control study	100	No	Ski slope or mild conductive HL	>6 months	No	Open ear canal HAs vs. classical HAs
Haab, 2019 [24]	Case control study	34	No	Mild to moderate hearing loss	>6 months	No	spectrally notched HAs group-unmodified HAs of the same type group
Henry, 2015 [25]	Case control study	30	>18 years	Symmetrical [difference between left and right ear (0.5, 1, 2, 4 kHz) pure-tone averages ≤15 dB HL] SNHL within the mild to moderately severe range (four-frequency pure-tone average 25–70 dB HL)	Not determined	No	HAs plus-noise (experimental) group-HAs only (control) group
Henry, 2017 [26]	RCT	55	No	PTA average (0.5, 1, 2, and 4 kHz) ~35–40 dB (mild to moderately severe hearing loss)	Not determined	No	HAs vs. HAs + Sound Generator
Hodgson, 2017 [27]	Single-blind crossover clinical trial	16	No	High-frequency audible SNHL	>6 months	No	RITE HAs with frequency compression group vs. RITE HAs without frequency compression
Jalilvand, 2015 [28]	Case control study	974	No	Unilateral or bilateral HL	Not determined	No	HAs vs. Noise Generator vs. both
McNeil, 2012 [29]	Retrospective case series study	70	No	from mild to severe (no further explanation)	Not determined	Yes	Group of patients with HL and tinnitus
Newman, 2012 [30]	Retrospective between-subject clinical study	56	No	Hearing levels not requiring amplification	Not determined	No	Neuromonics Tinnitus Treatment group-SG group
Ogut, 2012 [31]	Case Series	67	No	Any type of hearing loss	>3 months	No	HAs Tinnitus Masking Therapy (TMT) group
Oz, 2013 [32]	Double-Blinded RCT	21	No	Not determined	>6 months	Yes	betahistine and HA and/or a noise device vs. betahistine alone
Parazzini 2011 [33]	Case Control study	91	18–75 years	HL < 25 dB at 2 kHz and HL > 25 dB at frequencies higher than 2 kHz, bilateral symmetrical HL	>6 months	No	Tinnitus Retraining Therapy (TRT) with HA vs. TRT with sound generator
Peltier, 2012 [34]	Case Series	38	No	Unclear (considerable hearing loss at high frequencies)	Not determined	No	Linear octave frequency transposition (LOFT) hearing aid group
Radunz, 2019 [35]	RCT	33	>18 years	SNHL or mixed HL independent of degree and configuration	>3 months	No	Gingko biloba extract EGb 761 group vs. Beltone^®^ HA group vs. Gingko biloba plus HA group
Rocha, 2017 [36]	Case series	40	>18 years	Symmetrical bilateral mild to moderate SNHL	Not determined	Yes	HL and tinnitus group
Schaette, 2010 [37]	Case control study	114	No	SNHL or mixed HL	>3 months	Yes	HAs group vs. Noise device group
Searchfield, 2010 [38]	Case control study	58	No	SNHL	unclear	No	Counseling group vs. Counseling plus HAs
Searchfield, 2016 [39]	Study 2: Prototype evaluation	14	<70 years	Symmetrical mild-moderate HL	Not determined	No	“3D” masking group vs. “2D” masking group
	Study 3: Crossover pilot study	9	No	Mild-moderate SNHL in the fitting range	>6 months	No	TRT group vs. 3D masking group
Shabana, 2018 [40]	Case Control study	40	20–80 years	No more than 70 dB HL threshold in each ear	>6 months	No	HAs with Zen program activated vs. HAs without Zen program
Shekhawat, 2013 [41]	Case Series	25	No	Mild to moderate high-frequency sloping SNHL in the audiometric range of 0.25 to 8 kHz	>2 years	No	HA fitting group
Shekhawat, 2014 [42]	Double-blind, sham-controlled RCT	40	No	Sloping mild to severe sensorineural hearing loss	>2 years	No	real tDCS group vs. sham tDCS group
Shetty, 2019 [43]	Case control study	20	No	Bilateral, symmetrical, mild to severe SNHL	unclear	No	low pitch tinnitus group vs. high pitch tinnitus group
Strauss, 2017 [44]	Case control study	20	No	No	Not determined	No	BTE HAs group vs. notched environmental sound technology (NEST) HAs group
Sweetow, 2010 [45]	Case Series	16	No	Mild to moderately severe HL	>1 year	Yes	HA fitting master, fractal + master, fractal + master + noise, fractal alone group conditions
Tyler, 2017 [46]	Pilot Study	20	No	No more than 70 dB hearing loss from 250 to 4000 Hz	>4 months	No	HAs with zen program
Yakunina, 2019 [47]	Double-blinded RCT	94	>18 years	SNHL with PTA average of 250, 500, and 1000 Hz ≥ 25 dB HL, PTA of 2000, 4000, and 8000 Hz ≥ 40 dB, symmetric HL (difference between PTA of the right and left sides <15 dB HL)	Not determined	No	HAs with wide dynamic range compression group vs. HAs with frequency translation group vs. HAs with linear frequency transposition group
Yokota, 2020 [48]	Case Series	66	No	Not determined	Not determined	No	HAs group
Zarenoe, 2016 [49]	RCT	50	No	Mild-to-moderate SNHL	Not determined	No	Motivational Interviewing vs. HA fitting
Zarenoe, 2017 [50]	Case control study	92	No	Mild-to-moderate SNHL (PTA average) of 70 dB HL in both ears	Not determined	Yes	HL (2 subgroups with and without HA counseling) + tinnitus-HL but no tinnitus group

**Table 2:** Hearing aid fitting related methodological aspects.

ID	Software Used	Fitting Procedure Using Real Ear Measurement (REM)	Fitting Formula (Such as NAL, DSL etc.)	Use of Masking Sound (Type)	Counseling Regarding Tinnitus
Acar, 2014 [17]	Not determined	Not determined	Not determined	No	No
Araujo, 2016 [18]	Not determined	Not determined	Not determined	No	No
Berberian, 2017 [19]	Not determined	Not determined	Not determined	Individually calculated	No
Cabral, 2016 [20]	Not determined	Not determined	Not determined	No	No
Cribari, 2016 [21]	Not determined	Not determined	Not determined	No	No
dos Santos, 2014 [22]	EasyFit	Not determined	NAL-NL1	White noise	Yes
Forti, 2010 [23]	Not determined	Not determined	Not determined	No	Yes
Haab, 2019 [24]	Not determined	Not determined	Not determined	No	No
Henry, 2015 [25]	Not determined	Yes	NAL-NL2	Masking noise	Yes
Henry, 2017 [26]	Not determined	Yes	Manufacturer’s fitting formula (only option)	White noise, pink noise, and a spectrally shaped sound based on the user’s hearing loss	Yes
Hodgson, 2017 [27]	Audioscan Verift	Yes	DSL(I/O)] version 5.0	No	No
Jalilvand, 2015 [28]	Not determined	Yes	NAL-NL1	Noise generator	No
McNeil, 2012 [29]	Various	Yes	Various	No	No
Newman, 2012 [30]	Not determined	Not determined	Not determined	Sound Generator (SG), Neuromonics tinnitus treatment (NTT)	Yes
Ogut, 2012 [31]	NOAH-based custom programming	Not determined	Not determined	Band tailored masking sound	Yes
Oz, 2013 [32]	Not determined	Not determined	NAL-NL1	Wide-band noise	Yes
Parazzini, 2011 [33]	Not determined	Not determined	Not determined	SG	Yes
Peltier, 2012 [34]	Not determined	Not determined	Not determined	No	No
Radunz, 2020 [35]	Not determined	Not determined	Not determined	No	Yes
Rocha, 2018 [36]	OTO-Suite	Yes	NAL-NL1	SG	Yes
Schaette, 2010 [37]	Siemens Connexx	Not determined	NAL-NL1	Noise device	No
Searchfield, 2010 [38]	Not determined	Yes	Not determined	No	Yes
Searchfield, 2016 [39]	GN ReSound Aventa 2.0	Not determined	NAL NL 2	Rain sound	No
	GN ReSound Aventa 2.0	Not determined	DSL(I/O) v.5.0	Masking noise	Yes
Shabana, 2018 [40]	Compass version 5 on a NOAH 3 platform	Not determined	Not determined	Zen program	Yes
Shekhawat, 2013 [41]	WolverineTM Hybrid Jig with Inspiria Extreme	Yes	DSL(I/O) v5.0	No	No
Shekhawat, 2014 [42]	Not determined	Not determined	DSL(I/O)] v5.0	No	No
Shetty, 2019 [43]	NOAH or WINCHAP (v 3.00)	Yes	NAL-NL 2 or DSL (I/o) v5	Speech-shaped noise	No
Strauss, 2017 [44]	Not determined	Not determined	Not determined	Tailor made notch adjusted to the tinnitus frequency	No
Sweetow, 2010 [45]	Compass v4.542 beta software with NOAH link	Not determined	Not determined	Fractal tones, broadband noise	No
Tyler, 2017 [46]	Not determined	Not determined	Not determined	Zen program	Yes
Yakunina, 2019 [47]	Not determined	Not determined	Not determined	No	No
Yokota, 2020 [48]	Not determined	Not determined	Not determined	No	No
Zarenoe, 2016 [49]	Not determined	Not determined	Not determined	No	No
Zarenoe, 2017 [50]	Not determined	Not determined	Not determined	No	No

**Table 3:** Summary of research hypotheses, tinnitus assessment methods (tools, timeline) before and after HA fitting, overall study results.

ID	Research Hypothesis	Outcome Measures	Follow Up (Time)	Results
Acar, 2014 [17]	Hearing Aid (HA) fitting improves tinnitus perception	Tinnitus Handicap index (THI)	3 months	Significant improvement, even controlled by degree of hearing loss (HL)
Araujo, 2016 [18]	Improvement of tinnitus with HA usage and effect of tinnitus presence in HA satisfaction	THI, visual analog scale (VAS)	1 month after HA fitting, 3 months of effective use of HAs	Significant decrease of the THI at the end of the follow-up period
Berberian, 2017 [19]	HAs and maskers decrease tinnitus annoyance	THI, VAS	at least 6 months	Significant decrease of THI based on categorization (no actual scores provided)
Cabral, 2016 [20]	To assess the remission of emotional and auditory tinnitus impacts on users of hearing aids.	Tinnitus Acceptance Questionnaire (TAQ), Tinnitus Handicap Questionnaire (THQ)	3 months	Statistically significant improvement in both tinnitus domains after 3 months of HA usage
Cribari, 2016 [21]	Evaluate and qualify tinnitus in a group of elderly hearing aid wearers and determine the impact of symptoms on their quality of life. No baseline measurements were done	THI	Not determined	Recording was made only after HA fitting so no comparisons were feasible
dos Santos, 2014 [22]	Combined use of amplification and sound generator is more effective than amplification alone in reducing the discomfort of tinnitus	THI, VAS	3 months after fitting	No superiority of the combined use of amplification and sound generator over conventional amplification alone in reducing the discomfort of tinnitus.
Forti, 2010 [23]	Use of open ear canal HAs in tinnitus treatment	THI, VAS	9 months after fitting	Both groups showed improvement with regards to tinnitus (almost 50% according to THI). No significant differences between the two groups (open HAs and HAs). Control patients reported a lower comfort of use than OHA patients. No statistically significant correlations were found between THI or VAS among the different type of OHAs.
Haab, 2019 [24]	A tailor-made notch, individually adjusted to the tinnitus-frequency, in a hearing-aids amplification range	Tinnitus Questionnaire (TQ52)	3, 6 months	Differences between initial and final measurements differ in a statistically significant level between groups, in favor of the group using spectral masking.
Henry, 2015 [25]	Compare the use of combination instruments for tinnitus management with and without the use of broadband noise produced from the instruments.	Tinnitus Functional Index (TFI)	1–3 weeks HA adjustments, 3–4 months final evaluation	Both groups (control and experimental) revealed significant improvement based on reductions in mean TFI index scores. 26 of the 30 participants (86.7%) reported meaningful reduction in their tinnitus.
Henry, 2017 [26]	Relative efficacy of conventional receiver-in-the-canal hearing aids (HA), the same hearing aids with a sound generator (HA1SG), and extended-wear, deep fit hearing aids (EWHA)	TFI	1–3 weeks after fitting, 2 months after fitting, 4–5 months after fitting	All devices appear to offer clinically significant improvement in the functional effects of tinnitus but no statistical significance before-after, among devices or among groups was found
Hodgson, 2017 [27]	A crossover trial comparing FC to conventional wide dynamic range compression (WDRC) amplification in tinnitus patients.	TFI	6–8 weeks after fitting	Following the WDRC trial 44% of participants had tinnitus reduced by a clinically significant degree, only 19% achieved this in the FC trial. Wide dynamic range compression (WDRC) resulted in larger improvements in TFI and rating scale scores than when combined with FC across a group of tinnitus sufferers with high-frequency hearing loss and tinnitus.
Jalilvand, 2015 [28]	Comparison of hearing aid fitting and sound generator	patients’ satisfaction scale	1, 6, 12, 24 months after fitting	Amplification of sounds is effective in reducing or eliminating tinnitus loudness compared to noise generator.
McNeil, 2012 [29]	Hearing aids would be most effective when their frequency range encompassed an individual’ s tinnitus pitch.	TRQ	3 months after fitting	Clinically significant improvement in 51%. Total masking during HA use in Masking more common in low pitch tinnitus
Newman, 2012 [30]	To evaluate changes in perceived tinnitus handicap, following 6 months of sound therapy treatment using either Sound Generators (SGs) or Neuromonics Tinnitus Treatment	THI	1–6 months post fitting	No statistically significant differences were found between SGs and Neuromonics tinnitus treatment (NTT) at baseline or at the 6 months interval.
Ogut, 2012 [31]	Effect of tinnitus masking therapy (TMT) in our patient group in tinnitus	THQ, TRQ	4, 6, 8, 10, 12 weeks, 4, 5, 6 months, 8, 10, 12 months	Relief from annoyance was 55.9% and decrease of tinnitus effect on life was 67.2% at three months. Total rate for any degree of relief was 79.3% in normal hearing group, where in hearing-loss group it remained at 61.2%
Oz, 2013 [32]	Wide band differs from narrow band masker in terms of tinnitus	VAS, Mini-Tinnitus Questionnaire	3 months	No statistically significant differences between groups-however both showed significant improvements
Parazzini, 2011 [33]	TRT with HA vs. TRT with sound generator	THI, VAS	3, 6, 12 months	No significant differences between HA and sound masker
Peltier, 2012 [34]	Effect of linear octave frequency transposition (LOFT) hearing aid in tinnitus	VAS	Not determined	81% report long term tinnitus suppression
Radunz, 2020 [35]	Comparison between the effect of the use of the individual hearing aid, the use of Gingko biloba preparation and their combination	THI, VAS	90 days following treatment	Hearing aids were more effective in patients with shorter time to onset of tinnitus. G. biloba extract alone or in combination with the hearing aids was effective regardless of tinnitus duration.
Rocha, 2018 [36]	Real ear measurement (REM) is assistive to tinnitus treatment	THI, VAS	3, 6 months after initial evaluation	Significant decrease of THI in this group of patients
Schaette, 2010 [37]	Comparison of tinnitus suppression effects of conventional type HAs and frequency-lowering HAs in patients with HFHL	VAS, Tinnitus Questionnaire	1, 2, 3, 6 months after initial examination	There were no significant differences in primary or additional variables between hearing aid types at either 3 or 6 months.
Searchfield, 2010 [38]	Effect of HAs combined with counseling compared to counseling only	THQ	12 months post management	THQ scores were reduced following intervention but only the HA group scores were found to differ significantly. The percentage improvement in total THQ score for the HA group (37%) was approximately twice that of counseling alone (13%).
Searchfield, 2016 [39]	Study 2: Determine masking preferences amongst participants.	THI	2–2, 4 weeks	There was a significant difference in THI change between the 3D and center masking
	Study 3: Provide preliminary evidence of the effectiveness of spatial masking with counseling relative to a therapy using TRT principles.	TFI, Tinnitus Severity Numeric Scale (TSNS)	2–4 weeks, 2 months	The 3D scores reduced slightly more (11.78) than TRT (6.89) but the treatment by time interaction was not statistically significant
Shabana, 2018 [40]	Effectiveness of counseling and using amplification and sound stimulation (Zen tones of fractal music) technology	THI, TFI	After counseling, 4 months after HAs fitting	Statistically significant difference of THI scores between the post-counseling and following hearing aids fitting with or without Zen program, amount of improvement in the study group than in the control group except in THI emotional subscale score
Shekhawat, 2013 [41]	To examine the effects of high frequency modification of the DSL(I/O) v5.0 prescriptive procedure on short-term tinnitus perception.	TFI	Not determined	The higher the tinnitus pitch, the more the preferred real-ear output tended to match DSL(I/O) v5.0. For low- pitched tinnitus (< 4 kHz) the preferred output tended to be lower than that of DSL(I/O) v5.0 across the entire frequency range.
Shekhawat, 2014 [42]	To assess if combination of tDCS and hearing aids may facilitate priming of the brain for sound therapy resulting in greater hearing aid benefit in a shorter period of time.	Tinnitus Case History Questionnaire (TCHQ), TFI, TSNS, THQ, VAS	3 and 6 months following hearing aid fitting	The use of hearing aids led to a significant reduction in tinnitus handicap as measured with the TFI.
Shetty, 2019 [43]	(1) to determine the effect of gain adjustment on tinnitus perception in low and high pitch tinnitus groups (2) to compare SNR 50 using NAL NL 2 and DSL (I/o) v 5.0 fitting formulae in high and low pitch tinnitus groups and (3) to compare tinnitus relief data and SNR-50 scores pre- and post- hearing aid use.	THI	30 days after fitting	In the low pitch tinnitus group, the significantly lesser gain adjustment was noted in DSL (I/o) v5 (0.5) than NAL-NL 2 (1.83). Similarly, for the high pitch tinnitus group, gain adjustment required was significantly less using DSL (I/o) v5 (1.16 dB) compared to NAL-NL 2 (5.6 dB). Additionally, speech perception in noise was unaffected by the adjusted gain at tinnitus pitch using either NAL NL 2 or DSL (I/o) v5 prescriptive formulae.
Strauss, 2017 [44]	Proof-of-concept that tinnitus distress can be reduced by the notch-induced lateral inhibition in NEST	Tinnitus Questionnaire 12 (TQ12)	3 weeks post therapy	Both TQ12 and τ factor improvement more prominent in notched environmental sound technology (NEST) group (both groups improved though)
Sweetow, 2010 [45]	To determine if the presence of various acoustic stimuli delivered through a hearing aid would reduce short-term tinnitus annoyance, and lower the subjective tinnitus handicap.	THI, Tinnitus Reaction Questionnaire (TRQ), Tinnitus Annoyance Scale (TAS)	1, 3 and 6 months after fitting	The four fractal settings had similar median annoyance ratings, significantly better than the unaided (control) group TRQ: initial improvement, not consistent to 6 months. THI significant improvement at 6 months.
Tyler, 2017 [46]	Zen tones in the context of the Zen therapy are beneficial for tinnitus.	Tinnitus Primary Function Questionnaire (TPFQ), TFI, THQ, VAS	3, 6 months posttreatment	Statistically significant improvement in after 6 months (not right after HA fitting) in terms of TFI, VAS, TFPQ in a group of 20 patients fitted with Zen HAs
Yakunina, 2019 [47]	1. to isolate and evaluate the effects on tinnitus of HA alone without accompanying counseling or any other therapy 2. to investigate whether HAs provide long-term tinnitus suppression that lasts after cessation of their use 3. to explore how Frequency Lowering (FL) techniques (LFT and FT) performed compared with conventional WDRC in the same open-fit HA device in terms of tinnitus suppression for patients with high frequency hearing loss (HFHL)	THI, VAS awareness, VAS annoyance	3 months after fitting, 6 months after initial evaluation	HAs, with or without FL, seem to be effective for longer-term relief of tinnitus among patients with HFHL, and not only for the period of their use (3 months after)
Yokota, 2020 [48]	Effect of HA fitting in tinnitus	THI, VAS	1–12 months	Statistically significant improvement in all patients either with unilateral or bilateral tinnitus
Zarenoe, 2016 [49]	Effects of Motivational Interviewing (MI) as an adjunct to regular hearing aid fitting for patients with tinnitus and hearing loss.	THI	3 months after fitting	Both groups significantly decreased THI levels, hence the MI group showed statistically significant larger improvement
Zarenoe, 2017 [50]	Effect of hearing aids on memory tinnitus annoyance, capacity, sleep quality, hearing problems, speech recognition.	THI	3 months after fitting	Pre/post changes were significant for both groups on the Reading Span, PQSI and HHIE. The results of the THI revealed a significant improvement (*p* < 0.001) at follow-up for patients in the hearing loss and tinnitus matched group.

**Table 4:** Methodological limitations of the clinical studies included in this scoping review that may have a significant impact on the quality of results and their generalizability.

Limitations
Small or inadequate sample size Inadequate demographic inclusion/exclusion criteria (e.g., wide age range) Inadequate hearing-related inclusion/exclusion criteria (hearing thresholds range, tinnitus as primary complaint or not) Inadequate randomization or blinding Inadequate (short or non-clarified) follow-up timeline Inadequate selection of tinnitus assessment tools Unclarified tinnitus characteristics (e.g., vague tinnitus onset or tinnitus duration) Inadequate selection of primary and secondary endpoints for effect size assessment Inadequate study design (e.g., non-controlled)

## References

[1] Cima R.F.F., Mazurek B., Haider H., Kikidis D., Lapira A., Noreña A., Hoare D.J. (2019). A Multidisciplinary European Guideline for Tinnitus: Diagnostics, Assessment, and Treatment. Hno.

[2] Hesse G. (2016). Evidence and Evidence Gaps in Tinnitus Therapy. GMS Curr. Top. Otorhinolaryngol. Head Neck Surg..

[3] Bhatt J.M., Lin H.W., Bhattacharyya N. (2016). Tinnitus Epidemiology: Prevalence, Severity, Exposures And Treatment Patterns In The United States. JAMA Otolaryngol. Head Neck Surg..

[4] Oishi N., Shinden S., Kanzaki S., Saito H., Inoue Y., Ogawa K. (2011). Influence of Depressive Symptoms, State Anxiety, and Pure-Tone Thresholds on the Tinnitus Handicap Inventory in Japan. Int. J. Audiol..

[5] Ratnayake S.A.B., Jayarajan V., Bartlett J. (2009). Could an Underlying Hearing Loss Be a Significant Factor in the Handicap Caused by Tinnitus?. Noise Health.

[6] Schaette R., McAlpine D. (2011). Tinnitus with a Normal Audiogram: Physiological Evidence for Hidden Hearing Loss and Computational Model. J. Neurosci..

[7] Möhrle D., Hofmeier B., Amend M., Wolpert S., Ni K., Bing D., Klose U., Pichler B., Knipper M., Rüttiger L. (2019). Enhanced Central Neural Gain Compensates Acoustic Trauma-Induced Cochlear Impairment, but Unlikely Correlates with Tinnitus and Hyperacusis. Neuroscience.

[8] Guest H., Munro K.J., Plack C.J. (2019). Acoustic Middle-Ear-Muscle-Reflex Thresholds in Humans with Normal Audiograms: No Relations to Tinnitus, Speech Perception in Noise, or Noise Exposure. Neuroscience.

[9] Bramhall N., Beach E.F., Epp B., Le Prell C.G., Lopez-Poveda E.A., Plack C.J., Schaette R., Verhulst S., Canlon B. (2019). The Search for Noise-Induced Cochlear Synaptopathy in Humans: Mission Impossible?. Hear. Res..

[10] Karanicolas P.J., Farrokhyar F., Bhandari M. (2010). Blinding: Who, What, When, Why, How?. Can. J. Surg..

[11] Hall D.A., Smith H., Hibbert A., Colley V., Haider H.F., Horobin A., Londero A., Mazurek B., Thacker B., Fackrell K. (2018). The COMiT’ID Study: Developing Core Outcome Domains Sets for Clinical Trials of Sound-, Psychology-, and Pharmacology-Based Interventions for Chronic Subjective Tinnitus in Adults. Trends Hear..

[12] Tricco A.C., Lillie E., Zarin W., O’Brien K.K., Colquhoun H., Levac D., Moher D., Peters M.D.J., Horsley T., Weeks L. (2018). PRISMA Extension for Scoping Reviews (PRISMA-ScR): Checklist and Explanation. Ann. Intern. Med..

[13] Hopewell S., Clarke M., Lefebvre C., Scherer R. (2007). Handsearching versus Electronic Searching to Identify Reports of Randomized Trials. Cochrane Database Syst. Rev..

[14] Higgins J., Thomas J., Chandler J., Cumpston M., Li T., Page M., Welch V. (2021). Cochrane Handbook for Systematic Reviews of Interventions Version 6.2 (Updated February 2021). /handbook/current/chapter-21.

[15] McGowan J., Sampson M., Salzwedel D.M., Cogo E., Foerster V., Lefebvre C. (2016). PRESS Peer Review of Electronic Search Strategies: 2015 Guideline Statement. J. Clin. Epidemiol..

[16] Buscemi N., Hartling L., Vandermeer B., Tjosvold L., Klassen T.P. (2006). Single Data Extraction Generated More Errors than Double Data Extraction in Systematic Reviews. J. Clin. Epidemiol..

[17] Acar B. (2014). Effects of Hearing Aids on Tinnitus in Geriatric Patients with Age-Related Hearing Loss. Turk Geriatr. Derg..

[18] Araujo T.D.M., Iório M.C.M. (2016). Effects of Sound Amplification in Self-Perception of Tinnitus and Hearing Loss in the Elderly. Braz. J. Otorhinolaryngol..

[19] Berberian A.P., Ribas A., Imlau D., Guarinello A.C., Massi G., Tonocchi R., Riesemberg R., Martins J., Rosa M.R.D. (2017). Benefit of Using the Prosthesis with Sound Generators in Individuals with Tinnitus Associated With Mild to Moderately Severe Hearing Loss. Int. Tinnitus J..

[20] Cabral J., Tonocchi R., Ribas Â., Almeida G., Rosa M., Massi G., Berberian A.P. (2016). The Efficacy of Hearing Aids for Emotional and Auditory Tinnitus Issues. Int. Tinnitus J..

[21] Cribari J., Ribas A., Fonseca V.R., Moretti C.M., Zeigelboim B.S., Martins J., Rosa M.R.D. (2016). da Description of Tinnitus and Its Relation to Discomfort Level in a Group of Elderly Hearing Aid Wearers. Int. Tinnitus J..

[22] dos Santos G.M., Bento R.F., de Medeiros I.R.T., Oiticcica J., da Silva E.C., Penteado S. (2014). The Influence of Sound Generator Associated With Conventional Amplification for Tinnitus Control: Randomized Blind Clinical Trial. Trends Hear..

[23] Forti S., Crocetti A., Scotti A., Costanzo S., Pignataro L., Ambrosetti U., Del Bo L. (2010). Tinnitus Sound Therapy with Open Ear Canal Hearing Aids. B-ENT.

[24] Haab L., Lehser C., Corona-Strauss F.I., Bernarding C., Seidler H., Hannemann R., Strauss D.J. (2019). Implementation and Long-Term Evaluation of a Hearing Aid Supported Tinnitus Treatment Using Notched Environmental Sounds. IEEE J. Transl. Eng. Health Med..

[25] Henry J.A., Frederick M., Sell S., Griest S., Abrams H. (2015). Validation of a Novel Combination Hearing Aid and Tinnitus Therapy Device. Ear. Hear..

[26] Henry J.A., McMillan G., Dann S., Bennett K., Griest S., Theodoroff S., Silverman S.P., Whichard S., Saunders G. (2017). Tinnitus Management: Randomized Controlled Trial Comparing Extended-Wear Hearing Aids, Conventional Hearing Aids, and Combination Instruments. J. Am. Acad. Audiol..

[27] Hodgson S.-A., Herdering R., Singh Shekhawat G., Searchfield G.D. (2017). A Crossover Trial Comparing Wide Dynamic Range Compression and Frequency Compression in Hearing Aids for Tinnitus Therapy. Disabil. Rehabil. Assist. Technol..

[28] Jalilvand H., Pourbakht A., Haghani H. (2015). Hearing Aid or Tinnitus Masker: Which One Is the Best Treatment for Blast-Induced Tinnitus? The Results of a Long-Term Study on 974 Patients. Audiol. Neurootol..

[29] McNeill C., Távora-Vieira D., Alnafjan F., Searchfield G.D., Welch D. (2012). Tinnitus Pitch, Masking, and the Effectiveness of Hearing Aids for Tinnitus Therapy. Int. J. Audiol..

[30] Newman C.W., Sandridge S.A. (2012). A Comparison of Benefit and Economic Value between Two Sound Therapy Tinnitus Management Options. J. Am. Acad. Audiol..

[31] Ogut F., Mercan G.C., Ozturk K. (2012). Outcomes of Tinnitus Masking Therapy in Patients Selected Based on Audiological and Psychological Criteria. J. Int. Adv. Otol..

[32] Oz I., Arslan F., Hizal E., Erbek S.H., Eryaman E., Senkal O.A., Ogurlu T., Kizildag A.E., Ozluoglu L.N. (2013). Effectiveness of the Combined Hearing and Masking Devices on the Severity and Perception of Tinnitus: A Randomized, Controlled, Double-Blind Study. ORL.

[33] Parazzini M., Del Bo L., Jastreboff M., Tognola G., Ravazzani P. (2011). Open Ear Hearing Aids in Tinnitus Therapy: An Efficacy Comparison with Sound Generators. Int. J. Audiol..

[34] Peltier E., Peltier C., Tahar S., Alliot-Lugaz E., Cazals Y. (2012). Long-Term Tinnitus Suppression with Linear Octave Frequency Transposition Hearing Aids. PLoS ONE.

[35] Radunz C.L., Okuyama C.E., Branco-Barreiro F.C.A., Pereira R.M.S., Diniz S.N. (2020). Clinical Randomized Trial Study of Hearing Aids Effectiveness in Association with Ginkgo Biloba Extract (EGb 761) on Tinnitus Improvement. Braz. J. Otorhinolaryngol..

[36] Rocha A.V., Mondelli M.F.C.G. (2017). Sound Generator Associated with the Counseling in the Treatment of Tinnitus: Evaluation of the Effectiveness. Braz. J. Otorhinolaryngol..

[37] Schaette R., König O., Hornig D., Gross M., Kempter R. (2010). Acoustic Stimulation Treatments against Tinnitus Could Be Most Effective When Tinnitus Pitch Is within the Stimulated Frequency Range. Hear. Res..

[38] Searchfield G.D., Kaur M., Martin W.H. (2010). Hearing Aids as an Adjunct to Counseling: Tinnitus Patients Who Choose Amplification Do Better than Those That Don’t. Int. J. Audiol..

[39] Searchfield G.D., Kobayashi K., Hodgson S.-A., Hodgson C., Tevoitdale H., Irving S. (2016). Spatial Masking: Development and Testing of a New Tinnitus Assistive Technology. Assist. Technol..

[40] Shabana M.I., Dabbous A.O., Abdelmajeed M.A., Abdelkarim A.M.M. (2018). Counselling and Amplification with and without Fractal Music (Zen Tones) for Management of Patients Suffering from Hearing Loss and Tinnitus. Hear. Balance Commun..

[41] Shekhawat G.S., Searchfield G.D., Kobayashi K., Stinear C.M. (2013). Prescription of Hearing-Aid Output for Tinnitus Relief. Int. J. Audiol..

[42] Shekhawat G.S., Searchfield G.D., Stinear C.M. (2014). Randomized Trial of Transcranial Direct Current Stimulation and Hearing Aids for Tinnitus Management. Neurorehabilit. Neural Repair.

[43] Shetty H.N., Pottackal J.M. (2019). Gain Adjustment at Tinnitus Pitch to Manage Both Tinnitus and Speech Perception in Noise. J. Otol..

[44] Strauss D.J., Corona-Strauss F.I., Seidler H., Haab L., Hannemann R. (2017). Notched Environmental Sounds: A New Hearing Aid-Supported Tinnitus Treatment Evaluated in 20 Patients. Clin. Otolaryngol..

[45] Sweetow R.W., Sabes J.H. (2010). Effects of Acoustical Stimuli Delivered through Hearing Aids on Tinnitus. J. Am. Acad. Audiol..

[46] Tyler R., Cacace A., Stocking C., Tarver B., Engineer N., Martin J., Deshpande A., Stecker N., Pereira M., Kilgard M. (2017). Vagus Nerve Stimulation Paired with Tones for the Treatment of Tinnitus: A Prospective Randomized Double-Blind Controlled Pilot Study in Humans. Sci. Rep..

[47] Yakunina N., Lee W.H., Ryu Y.-J., Nam E.-C. (2019). Tinnitus Suppression Effect of Hearing Aids in Patients With High-Frequency Hearing Loss: A Randomized Double-Blind Controlled Trial. Otol. Neurotol..

[48] Yokota Y., Yamashita A., Koyama S., Kitano K., Otsuka S., Kitahara T. (2020). Retrospective Evaluation of Secondary Effects of Hearing Aids for Tinnitus Therapy in Patients with Hearing Loss. Auris Nasus Larynx.

[49] Zarenoe R., Söderlund L.L., Andersson G., Ledin T. (2016). Motivational Interviewing as an Adjunct to Hearing Rehabilitation for Patients with Tinnitus: A Randomized Controlled Pilot Trial. J. Am. Acad. Audiol..

[50] Zarenoe R., Hallgren M., Andersson G., Ledin T. (2017). Working Memory, Sleep, and Hearing Problems in Patients with Tinnitus and Hearing Loss Fitted with Hearing Aids. J. Am. Acad. Audiol..

[51] Haider H., Fackrell K., Kennedy V., Hall D.A. (2016). Dimensions of Tinnitus-Related Complaints Reported by Patients and Their Significant Others: Protocol for a Systematic Review. BMJ Open.

[52] Hall D.A., Hibbert A., Smith H., Haider H.F., Londero A., Mazurek B., Fackrell K. (2019). One Size Does Not Fit All: Developing Common Standards for Outcomes in Early-Phase Clinical Trials of Sound-, Psychology-, and Pharmacology-Based Interventions for Chronic Subjective Tinnitus in Adults. Trends Hear..

[53] Tyler R.S., Coelho C., Noble W. (2006). Tinnitus: Standard of Care, Personality Differences, Genetic Factors. ORL J. Otorhinolaryngol. Relat. Spec..

[54] Hoare D.J., Edmondson-Jones M., Sereda M., Akeroyd M.A., Hall D. (2014). Amplification with Hearing Aids for Patients with Tinnitus and Co-Existing Hearing Loss. Cochrane Database Syst. Rev..

